# Increased serum adenosine deaminase activity in patients with adult-onset Still's disease

**DOI:** 10.1186/s12865-022-00477-5

**Published:** 2022-01-29

**Authors:** Zhiye Xu, Linyu Geng, LiLi Guo, Hongyan Song, Jie Pan, Han Shen, Sen Wang

**Affiliations:** 1 Department of Clinical Laboratory Medicine, The Affiliated Drum Tower Hospital of Nanjing University Medical School, Nanjing, 210008 China; 2 Department of Rheumatology and Immunology, The Affiliated Drum Tower Hospital of Nanjing University Medical School, Nanjing, 210008 China

**Keywords:** AOSD, ADA, Diagnosis, Disease activity

## Abstract

**Background:**

Adult-onset Still's disease (AOSD) is a systemic inflammatory disease of unknown etiology, lacking specific diagnosis and disease activity evaluation indicators. This study will analyze the activity and clinical significance of Adenosine deaminase (ADA) in AOSD patients.

**Methods:**

Totally 53 AOSD patients, 60 patients with other autoimmune diseases including systemic lupus erythematosus (SLE), sjogren syndrome (SS) and rheumatoid arthritis (RA), as well as 60 healthy subjects were included in this study. AOSD activity was determined by Pouchot score. We analyzed the correlation between ADA activity and clinical parameters. In addition, the correlation between ADA activity and disease activity score was also analyzed.

**Results:**

This study showed that the activity of ADA in AOSD patients was significantly higher than that of healthy controls, SLE, SS and RA patient groups (*p* < 0.0001). The ADA activity of AOSD patients decreased significantly after systemic treatment (*p* < 0.0001). Correlation analysis showed that ADA activity was positively correlated with ALT(*r* = 0.54, *p* < 0.0001), AST (*r* = 0.82, *p* < 0.0001) and serum ferritin (*r* = 0.67, *p* < 0.001). ADA activity was negatively correlated with white blood cell (*r* = − 0.42, *p* = 0.002) and platelet counts (*r* = − 0.44, *p* = 0.001). We also found a significant positive correlation between the activity of ADA and Pouchot score in AOSD patients (*r* = 0.51, *p* = 0.001). Receiver operating characteristic (ROC) curve analysis showed that ADA activity had a sensitivity of 93.3%, and a specificity of 83% for the diagnosis of AOSD, with an area under the curve of 0.93.

**Conclusion:**

This study showed that serum ADA activity can be used as a potential biomarker for AOSD diagnosis and disease activity assessment.

## Introduction

Adult-onset Still's disease (AOSD) is a systemic inflammatory disease with low incidence rate and unclear etiology. The main manifestations of AOSD patients include fever, rash, sore throat, and joint pain, as well as abnormal laboratory tests, such as peripheral leukocytosis, abnormal liver cell function, and increased serum ferritin [1, 2]. The pathogenesis of AOSD is still unclear, and it had been revealed to be caused by infection, genetic factors, cytokine-mediated inflammation, and abnormal immune regulation caused by apoptosis [3, 4]. Patients with AOSD have different clinical manifestations, lacking specific diagnostic indicators and clinical features. The diagnosis of AOSD mainly depends on exclusion of other autoimmune diseases. AOSD disease activity mainly refers to serum ferritin level, white blood cell count and some clinical manifestations [5, 6]. The Pouchot score had been used to evaluate AOSD activity in some studies [7, 8].

Adenosine deaminase (ADA) is an important enzyme of purine nucleoside metabolism which is widely distributed in various tissues and cells of the human body. Damage to hepatocytes can cause ADA to be released into the circulation, and serum ADA activity is a sensitive indicator of liver damage [9]. Therefore, ADA activity is an indicator of hepatocyte function and can be used as one of the routine items in liver function tests. ADA is also an important decomposing enzyme in the adenosine metabolism pathway and plays a key role in regulating the level of intracellular and extracellular adenosine levels. ADA affects the immune function of adenosine by regulating the level of extracellular adenosine, and indirectly exerting its immune regulation function [10, 11]. In addition, ADA is essential for development and maturation of lymphocytes, independent of enzyme activity, and plays an important regulatory role in proliferation, differentiation, and immune regulation [12]. Increased ADA activity has been observed in some immune-related diseases, such as inflammation, tumor, and autoimmune diseases [13–15]. ADA activity can be elevated in serum and pleural fluid of tuberculosis patients. ADA activity, especially pleural effusion, is a sensitive indicator for diagnosis of tuberculous pleurisy [16–18]. Recently, elevated levels of ADA in sera have been described in autoimmune diseases, including SLE and juvenile rheumatoid arthritis (JIA) [19, 20]. Further, plasma ADA2 activity can be used as a biomarker for macrophage activation syndrome in systemic JIA patients [21]. Although systemic JIA and AOSD patients share similar clinical manifestations, pathogenesis, and treatment response, little is known about the level and biology of ADA activity in AOSD patients. In this study, we analyzed the activity of ADA in the serum of AOSD patients, analyzed its correlation with disease activity, and further studied whether ADA activity is suitable as a marker for AOSD diagnosis and activity evaluation.

## Materials and methods

### Patients and control participants

We retrospectively analyzed laboratory, clinical, and treatments data of 53 AOSD patients initially admission at the Nanjing Drum Tower Hospital from January 2019 to December 2020. All of these patients met the Yamaguchi criteria [22]. 60 patients with other autoimmune diseases including SLE, sjogren syndrome (SS) and rheumatoid arthritis (RA), as well as 60 age- and gender-matched healthy subjects as controls were also included in this study. We collect clinical data from the electronic medical record system in a standardized form, including laboratory data, demographics, and clinical symptoms. Demographic and clinical characteristics of these patient samples are presented in Table 1. The disease activity assessment of AOSD patients uses the Pouchot score [8]. The scoring standard includes 12 items, including fever, skin rashes, sore throat, arthritis, myalgia, pleuritis, pericarditis, pneumonitis, lymphadenopathy, hepatomegaly or abnormal liver function tests, elevated leukocyte count, each item was 1 point.

**Table 1:** Clinical characteristics and laboratory findings of the study subjects

Characteristics	AOSD	SLE	SS	RA	Healthy control	P value*
Age (mean ± SD)	39.5 ± 16.1	41.3 ± 16.4	42.9 ± 13.3	39.7 ± 6.8	38.5 ± 10.1	0.69
Gender (male/female)	13/40	13/47	14/46	13/47	14/46	–
*Clinical manifestations*						
Fever	46 (87%)	19 (32%)	12 (20%)	4 (7%)	–	–
Skin rash	37 (70%)	32 (53%)	12 (20%)	4 (7%)	–	–
Arthritis	43 (81%)	12 (20%)	18 (30%)	60 (100%)	–	–
Sore throat	25 (47%)	0 (0%)	0 (0%)	2 (3%)	–	–
Hepatomegaly or abnormal liver	44 (83%)	4 (7%)	7 (12%)	7 (12%)	–	–
Function tests Splenomegaly	21 (40%)	4 (7%)	7 (12%)	7 (12%)	–	–
Lymphadenopathy	9 (17%)	6 (10%)	6 (10%)	6 (10%)	–	–
Pleuritis	11 (21%)	15 (25%)	2 (3%)	4 (7%)	–	–
Pericarditis	12 (21%)	8 (13%)	0 (0%)	3 (5%)	–	–
Pneumonitis	16 (30%)	9 (15%)	17 (28%)	5 (8%)	–	–
Abdominal pain	3 (6%)	3 (6%)	0 (0%)	0 (0%)	–	–
Elevated leukocyte count	34 (64%)	10 (17%)	5 (8%)	9 (15%)	–	–
*Laboratory findings*						
WBC (10^9^/L)	13.29 ± 6.29	6.38 ± 3.19	6.15 ± 4.55	6.44 ± 3.29	6.21 ± 1.38	< 0.0001
Neutrophils (10^9^/L)	11.43 ± 6.27	4.89 ± 2.74	4.53 ± 4.18	4.45 ± 2.90	3.61 ± 1.04	< 0.0001
AST (IU/L)	58.57 ± 88.34	18.88 ± 12.71	27.12 ± 23.65	26.78 ± 34.34	23.01 ± 15.15	< 0.003
ALT (IU/L)	78.96 ± 100.4	20.13 ± 9.70	26.83 ± 28.40	33.37 ± 66.80	29.19 ± 15.39	< 0.0002
LDH (IU/L)	531.5 ± 360.1	288.3 ± 88.97	265.3 ± 151.3	187.3 ± 49.1	175.0 ± 29.43	< 0.0001
Ferrition (ng/mL)	4601 ± 6208	655.9 ± 457.5	533.3 ± 642.3	293.9 ± 390.7	200.0 ± 158.2	< 0.0001
ESR (mm/h)	55.08 ± 30.41	42.58 ± 32.22	37.87 ± 31.06	34.8 ± 24.5	7.34 ± 5.83	< 0.0001
CRP (mg/dL)	60.21 ± 40.86	17.44 ± 22.19	12.83 ± 20.81	16.01 ± 23.36	3.91 ± 2.02	< 0.0001
Pouchot score	5.58 ± 1.98	–	–	–	–	–

Data was represented by the mean ± standard deviation

“*” indicates differences between AOSD patients and healthy controls

### Haematology analysis

The serum ADA activity, transaminase and C-reactive protein (CRP) of patients and healthy controls were detected using a biochemical analyzer (Beckman, Germany). White blood cell count, platelet count, hemoglobin test using blood analyzer (Sysmex Corporation, Japan). Erythrocyte sedimentation rate (ESR) was detected using an erythrocyte sedimentation rate analyzer (Vital diagnostinic, Italy). The detection of serum ferritin uses electrochemiluminescence immunoassay analyzer (Roche, Switzerland). These data were obtained from the hospital's laboratory information system and medical record system.

### Receiver operating characteristic (ROC) curve analysis

The ROC curve was generated based on the serum ADA activity of AOSD patients and healthy controls. The Youden index was used to determine the best cut-off value for the difference between AOSD patients and healthy controls. The index was defined as the sum of sensitivity and specificity minus one. The Youden index was defined as the sum of sensitivity and specificity minus one. The overall diagnostic accuracy was evaluated based on the area under the ROC curve (AUC).

### Statistical analysis

The measurement data was represented by the mean ± standard deviation. The unpaired Student's *t* test was used to compare the difference between patients and controls, and the Wilcoxon signed-rank test was used to compare the patients before and after treatment. *p* < 0.05 was considered as statistically significant difference between the two groups. Pearson correlation analysis was used to study the correlation between the indicators. Statistical analysis and graphing were done using GraphPad Prism5 software.

## Results

### Serum adenosine deaminase is significantly elevated in patients with adult-onset still's disease

**Fig. 1:**
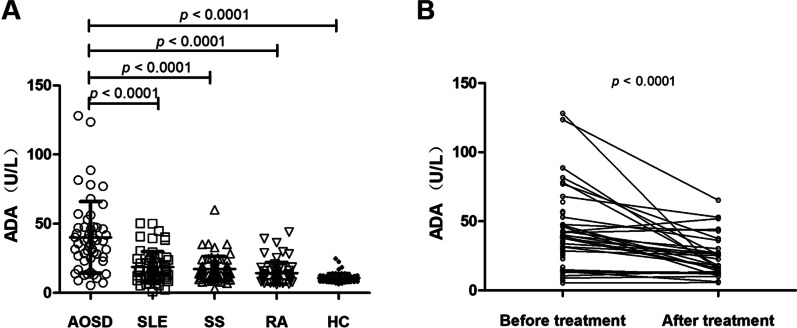
Serum adenosine deaminase in patients with AOSD was significantly elevated. **a** Comparison of serum ADA activity in different groups of patients. Data are expressed as mean ± standard deviation. **b** Comparison of serum ADA activity before and after treatment in AOSD patients. Statistical analysis using paired T test

We first analyzed the differences in serum adenosine deaminase activity in AOSD patients, SLE patients, RA patients, SS patients and healthy controls. The results showed that the serum ADA activity in AOSD patients was significantly higher than that of healthy controls (40.17 ± 25.88 *vs* 10.79 ± 3.40, *p* < 0.0001), SLE (40.17 ± 25.88 *vs* 18.73 ± 1.44, *p* < 0.0001), RA (40.17 ± 25.88 *vs* 17.32 ± 9.04, *p* < 0.0001) and SS patients (40.17 ± 25.88 *vs* 14.28 ± 7.96, *p* < 0.0001) (Fig. 1a). We also analyzed the ADA activity of patients when they were admitted to the hospital, and one month after they were discharged. We found that, after systemic immunosuppressant treatment, the serum ADA activity of AOSD patients decreased significantly (*p* < 0.0001) (Fig. 1b).

### Correlation analysis serum ADA activity and transaminase activity

**Fig. 2:**
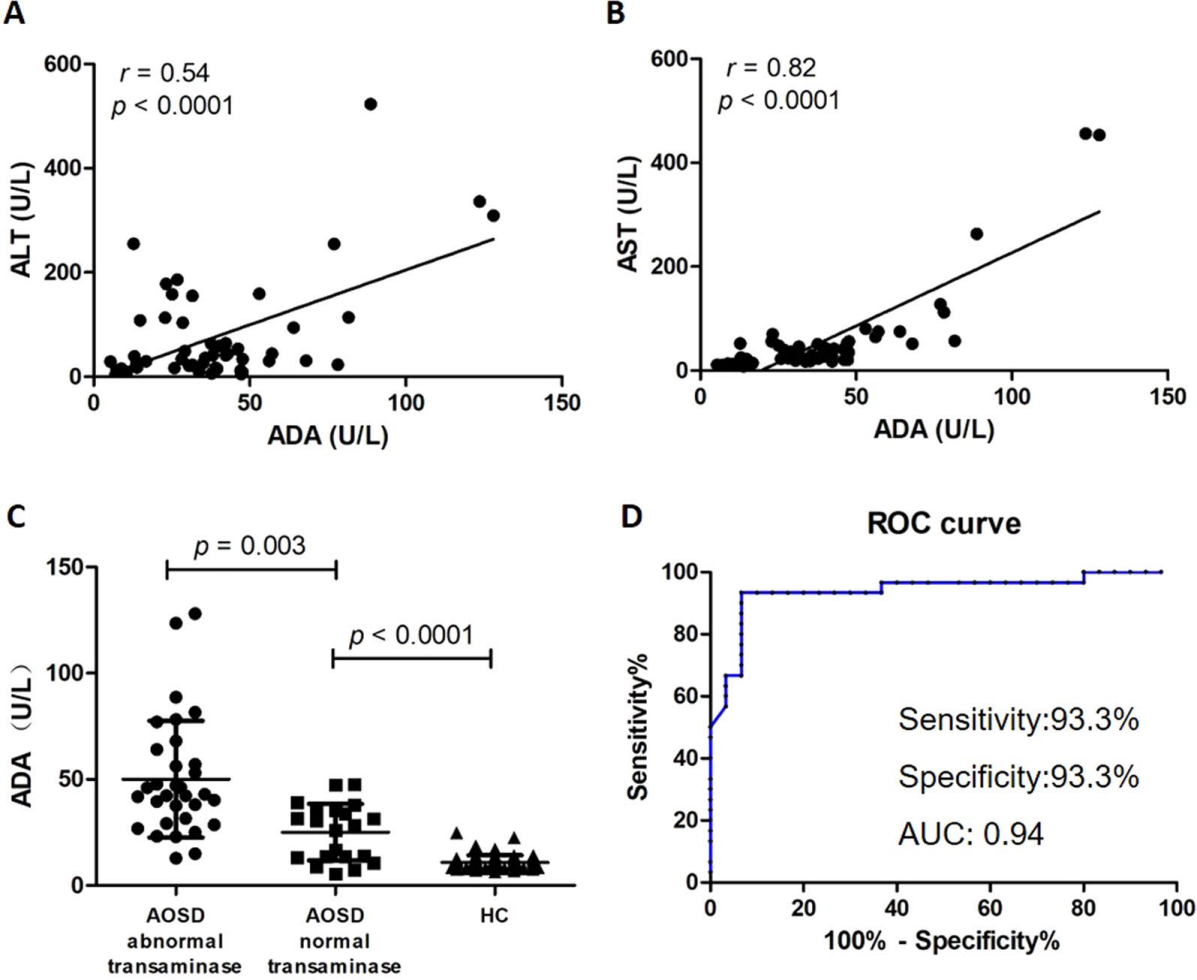
Correlation analysis serum ADA activity and transaminase activity. Correlation between ADA activity and ALT/AST (**a, b**). Comparison of ADA activity in AOSD patients with abnormal transaminase group (ALT/AST > 40 U/L), AOSD patients with and normal transaminase group and healthy control group (**c**). ROC curve analysis of ADA activity to distinguish AOSD related liver dysfunction and non-AOSD related liver dysfunction. Cut off value: 21.45 U/L. Sensitivity:93.3%. Specificity: 93.3%. AUC: 0.94 (**d**)

As serum ADA activity is a sensitive indicator of liver damage, we analyzed the correlation between ADA activity and ALT/AST, and found that ADA activity was correlated with both ALT(*r* = 0.54, *p* < 0.0001) and AST (*r* = 0.82, *p* < 0.0001) (Fig. 2a, b). We analyzed the difference in serum ADA activity between patients with abnormal transaminase and normal transaminase, and found that AOSD patients with normal transaminase had significantly lower ADA activity than patients with abnormal transaminase (25.10 ± 2.91 *vs* 50.06 ± 4.86, *p* = 0.003) but significantly higher than healthy controls (25.10 ± 2.91 *vs* 10.79 ± 0.46, *p* < 0.0001) (Fig. 2c). According to the ROC curve analysis, the sensitivity and specificity of ADA activity as a diagnostic marker to distinguish AOSD related liver dysfunction and non-AOSD related liver dysfunction were both 93.3%, and area under the ROC curve was 0.94 (Fig. 2d).

### Correlation analysis between ADA activity and disease activity

**Fig. 3:**
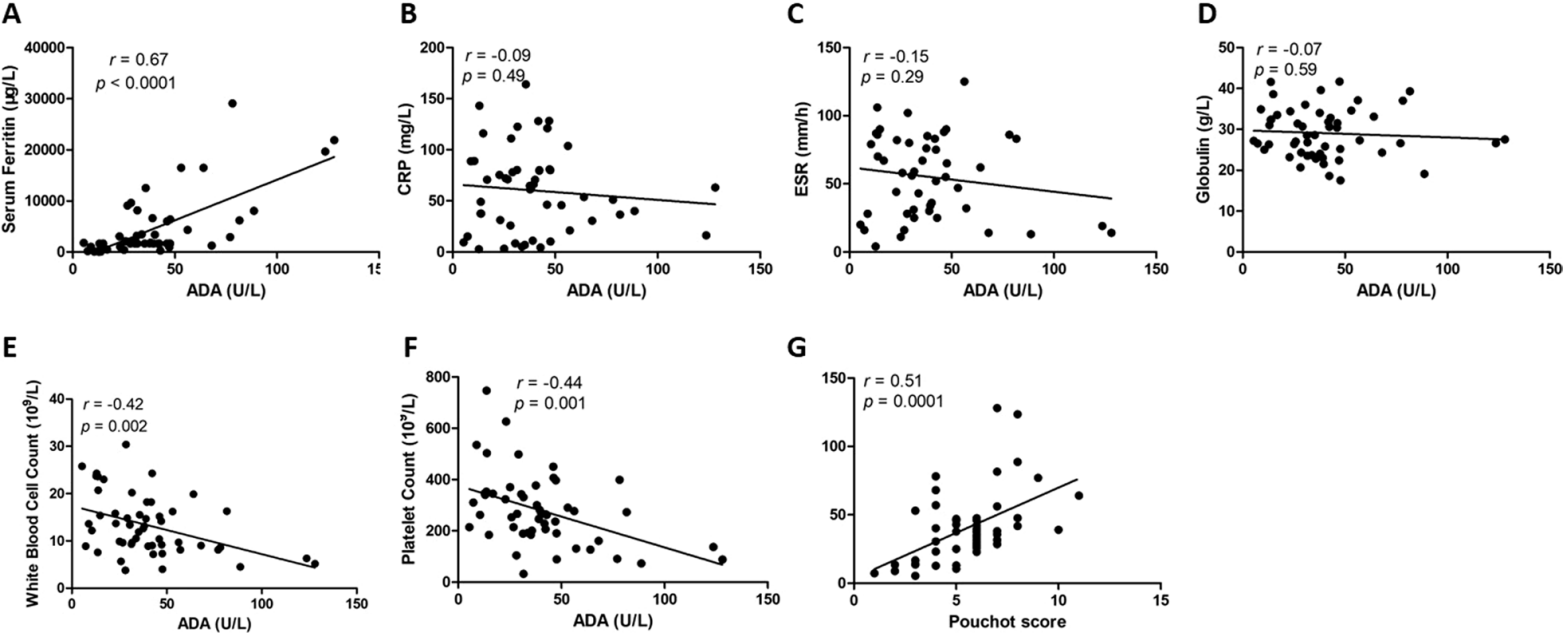
Correlation analysis between ADA activity and laboratory indicators. (**a–f**) Correlation of serum ADA activity with Serum ferritin, CRP, ESR, Globulin, WBC count and platelet count. Correlation between serum ADA activity and Pouchot AOSD activity score (**g**)

In order to explore whether serum ADA activity was related to the disease activity in AOSD patients, we analyzed the correlation between serum ADA activity and laboratory hematology data. The results revealed that serum ADA activity was significantly positively correlated with serum ferritin (*r* = 0.67, *p* < 0.0001) but not with CRP, ESR, and globulin (Fig. 3a–d). In addition, we found that serum ADA activity was negatively correlated with white blood cell count (*r* = − 0.42, *p* = 0.002) and platelet count (*r* = − 0.44, *p* = 0.001) (Fig. 3e, f). We scored each patient and further analyzed the correlation between serum ADA activity and Pouchot score. The analysis results found that serum ADA activity was significantly and positively correlated with the Pouchot score (*r* = 0.51, *p* = 0.0001) (Fig. 3g).

### The correlation between clinical manifestations and ADA activity in patients with AOSD

**Fig. 4:**
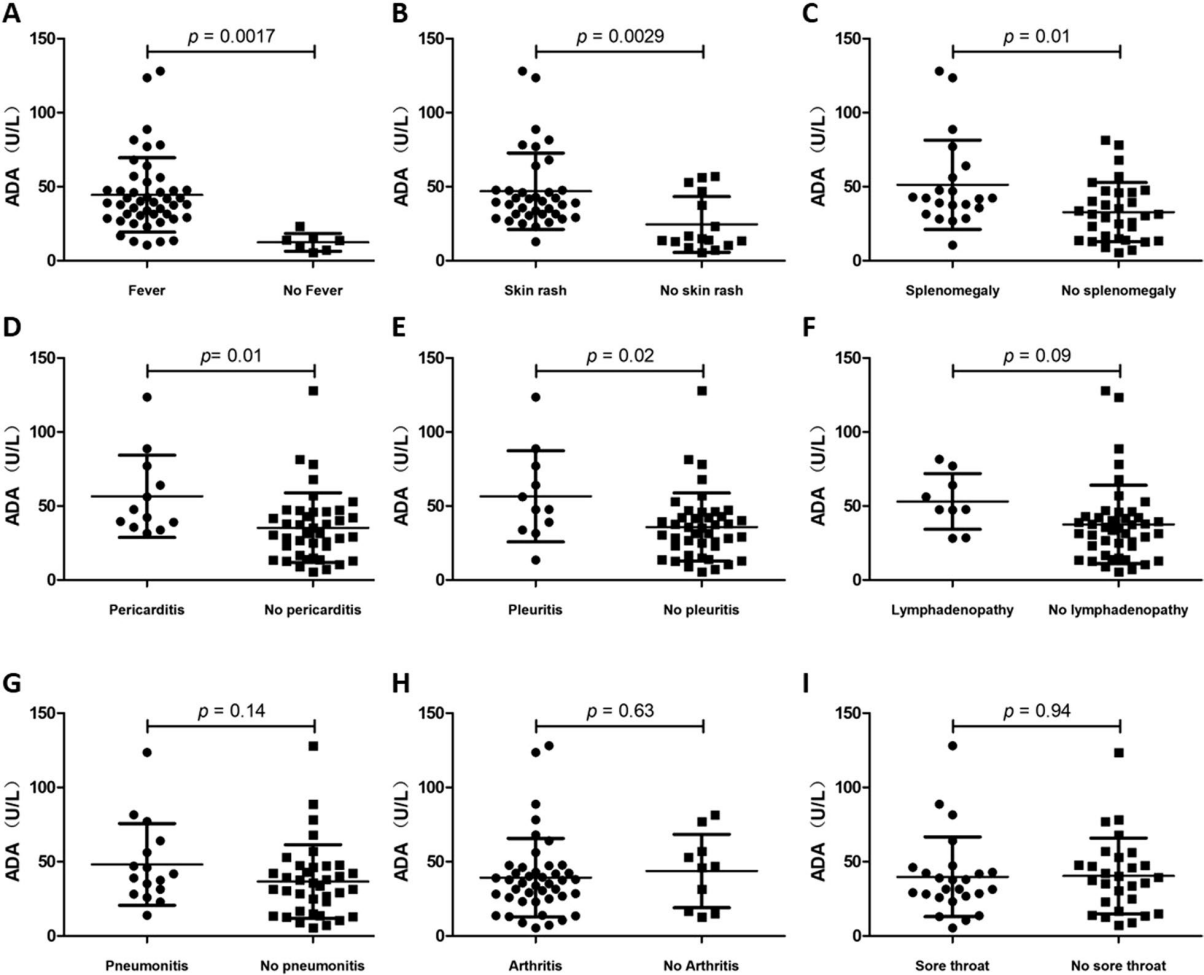
The correlation of ADA activity with clinical manifestations. ADA activity was significantly increased in patients with fever, skin rash, splenomegaly, pleuritis, and pericarditis (**a–e**). The activity of ADA was not statistically correlated with clinical manifestations such as lymphadenopathy, pneumonitis, arthritis, and sore throat (**f–i**)

We also analyzed the correlation of ADA activity with fever, skin rash, Lymphadenopathy and other clinical manifestations. The results showed that ADA activity was significantly increased in patients with fever (44.39 ± 25.13 *vs* 12.47 ± 5.97, *p* = 0.0017), skin rash (46.95 ± 25.77 *vs* 24.51 ± 18.78, *p* = 0.0029), splenomegaly (51.30 ± 30.15 *vs* 32.87 ± 19.96, *p* = 0.01), pleuritis (56.62 ± 30.75 *vs* 35.86 ± 22.96, *p* = 0.016), and pericarditis (56.59 ± 27.73 *vs* 35.37 ± 23.55, *p* = 0.01) (Fig. 4a–e). There was no statistical correlation between ADA activity and clinical manifestations such as lymphadenopathy, pneumonitis, arthritis, and sore throat (Fig. 4f–i).

### ROC curve analysis of serum ADA activity for diagnosis of AOSD

**Fig. 5:**
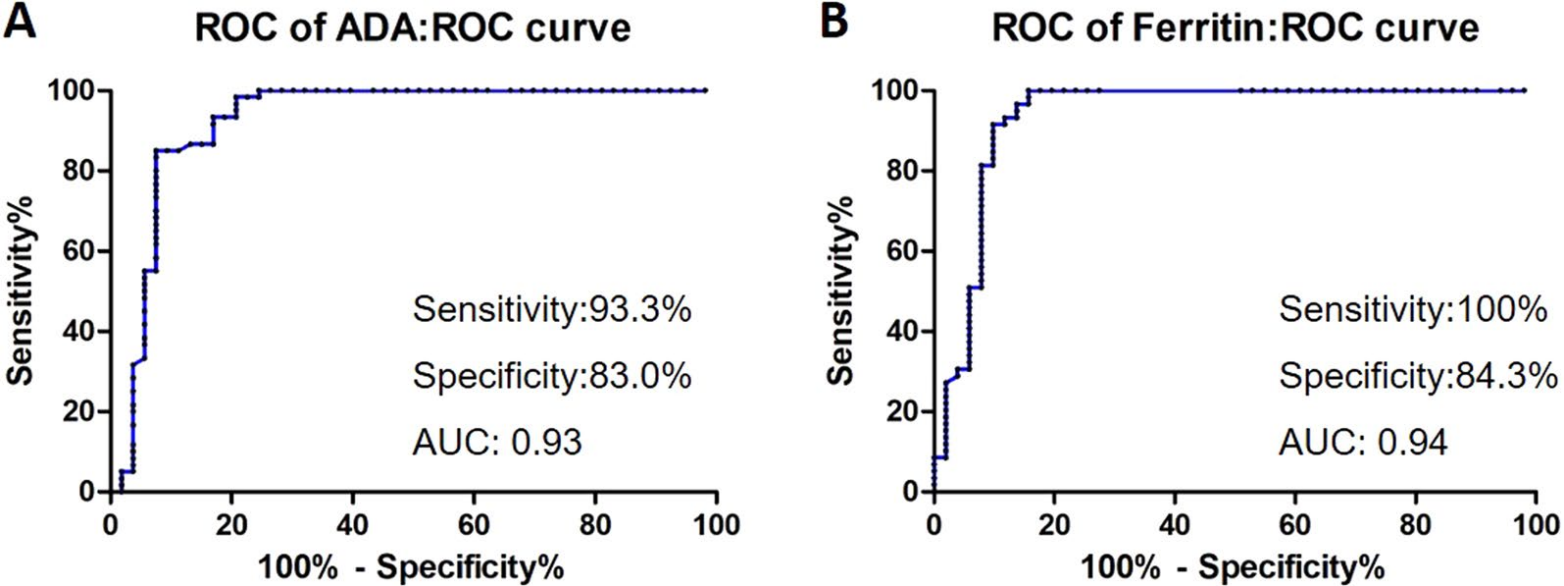
ROC curve analysis of serum ADA activity and serum ferritin for diagnosis of AOSD. Cut off value: 14.5 U/L. Sensitivity: 93.3%. Specificity: 83.0%. AUC: 0.93 (**a**). Cut off value: 759.9 μg/L. Sensitivity: 100%. Specificity:84.3%. AUC: 0.94 (**b**)

We analyzed the accuracy of using serum ADA activity as a diagnostic indicator to distinguish AOSD patients from healthy controls. According to the ROC curve analysis, we used Youden Index to determine the cut off value of 14.5 U/L, sensitivity of ADA activity for diagnosing AOSD was 93.3%, specificity was 83.0%, and area under the ROC curve was 0.93 (Fig. 5a). The diagnostic performance of ADA activity is almost as good as serum ferritin, and the area under the ROC curve for serum ferritin to distinguish AOSD patients from healthy controls is 0.94 (Fig. 5b).This indicates that serum ADA activity may be a potential diagnostic marker for AOSD.

## Discussion

Patients with AOSD have different clinical manifestations, and lack specific diagnostic indicators and clinical features. This study explored the role of serum ADA activity in AOSD patients. We found that ADA activity was significantly increased in the serum of AOSD patients, and also significantly decreased after one month of immunosuppressant treatment. ROC curve analysis showed that, the use of serum ADA activity to diagnose AOSD had high sensitivity and specificity. The activity of serum ADA in AOSD patients was significantly positively correlated with serum ferritin, and negatively correlated with white blood cell count. In addition, ADA activity was significantly positively correlated with the Pouchot score.

There was a study that showed ADA activity may be a potential marker for the diagnosis of AOSD [23]. However, this study included fewer cases and did not analyze the relationship between ADA activity and disease activity. We found that serum ADA activity was significantly increased in the serum of AOSD patients, and may be used as a potential marker for the diagnosis of AOSD, which is consistent with previous reports. We also found that the activity of ADA had a significant positive correlation with serum ferritin, but a significant negative correlation with white blood cell count and platelet count. This was consistent with previous report by Ruscitti et al., who showed that ferritin was negatively correlated with white blood cell count in AOSD patients [24]. One possible reason for the negative correlation between serum ADA activity and white blood cell count was that the destruction of white blood cells can release ADA into the circulation. Serum ADA activity was related to the Pouchot score of AOSD patients, and the correlation was higher than that of serum ferritin, indicating that ADA activity is also a potential marker for assessing disease activity.

Adenosine deaminase is a key enzyme in the adenosine catabolism pathway that can affect the immune function of adenosine by regulating the level of extracellular adenosine. Adenosine is considered to be an immunosuppressive molecule that can avoid excessive inflammation [11, 25, 26]. The increased activity of ADA reduces the concentration of adenosine in the peripheral circulation. Therefore, the association between serum ADA activity and disease activity in AOSD patients may be due to the fact that increased ADA activity reduces the concentration of adenosine, which leads to excessive activation of immune cells. ADA has two subtypes, ADA1 and ADA2, of which ADA2 is the main form present in human plasma. One study suggests that ADA2 activity can be used as a biomarker for macrophage activation syndrome in patients with systemic JIA [21]. The ADA activity measured in this study refers to the total ADA, and the specific type of ADA plays a role in AOSD patients needs to be further studied. The ADA activity in AOSD patients with normal ALT and AST was still significantly higher than that in healthy controls, indicating that serum ADA is possibly produced and released by other cells besides hepatocyte damage. Abnormal function or damage of peripheral blood immune cells in AOSD patients may be one of the reasons for the increased serum ADA activity. The causes and effects of increased ADA activity in serum of AOSD patients still need to be further studied.

Limitations of the study are as followings: Firstly, the number of AOSD patients is relatively small, a large sample size study is still needed to verify the findings of this study. In addition, this study is a retrospective study, and a prospective cohort study would be beneficial to understand the clinical application value and to evaluate potential biological mechanisms of ADA activities in AOSD patients.

## Conclusions

In summary, this study found that serum ADA activity was significantly increased in AOSD patients, and its level was related to serum ferritin and disease activity. Serum ADA activity may thus be used as a potential biomarker for the diagnosis and activity evaluation of AOSD.

## Data Availability

The datasets used and analyzed during the current study are available from the corresponding author on reasonable request.
